# A Doubling of Microphytobenthos Biomass Coincides with a Tenfold Increase in Denitrifier and Total Bacterial Abundances in Intertidal Sediments of a Temperate Estuary

**DOI:** 10.1371/journal.pone.0126583

**Published:** 2015-05-11

**Authors:** Helen Decleyre, Kim Heylen, Koen Sabbe, Bjorn Tytgat, Dieter Deforce, Filip Van Nieuwerburgh, Carl Van Colen, Anne Willems

**Affiliations:** 1 Laboratory of Microbiology (LM-UGent), Department of Biochemistry and Microbiology, Ghent University, Ghent, Belgium; 2 Laboratory of Protistology and Aquatic Ecology, Department of Biology, Ghent University, Ghent, Belgium; 3 Laboratory of Pharmaceutical Biotechnology, Faculty of Pharmaceutical Sciences, Ghent University, Ghent, Belgium; 4 Marine Biology Research Group, Department of Biology, Ghent University, Ghent, Belgium; Wageningen University, NETHERLANDS

## Abstract

Surface sediments are important systems for the removal of anthropogenically derived inorganic nitrogen in estuaries. They are often characterized by the presence of a microphytobenthos (MPB) biofilm, which can impact bacterial communities in underlying sediments for example by secretion of extracellular polymeric substances (EPS) and competition for nutrients (including nitrogen). Pyrosequencing and qPCR was performed on two intertidal surface sediments of the Westerschelde estuary characterized by a two-fold difference in MPB biomass but no difference in MPB composition. Doubling of MPB biomass was accompanied by a disproportionately (ten-fold) increase in total bacterial abundances while, unexpectedly, no difference in general community structure was observed, despite significantly lower bacterial richness and distinct community membership, mostly for non-abundant taxa. Denitrifier abundances corresponded likewise while community structure, both for *nirS* and *nirK* denitrifiers, remained unchanged, suggesting that competition with diatoms for nitrate is negligible at concentrations in the investigated sediments (appr. 1 mg/l NO_3_^-^). This study indicates that MPB biomass increase has a general, significantly positive effect on total bacterial and denitrifier abundances, with stimulation or inhibition of specific bacterial groups that however do not result in a re-structured community.

## Introduction

The rate of terrestrial nitrogen input has more than doubled in the past century, mostly through fossil fuel combustion and increased use of agricultural fertilizers [1, 2]. When it is not removed by biotic uptake or dissimilatory nitrate reduction in streams and rivers, excessive, anthropogenically-derived nitrogen ends up in estuaries and coastal areas [3], where it is implicated in eutrophication [4] that can generate an excessive biochemical oxygen demand resulting in hypoxic zones [5], and can promote harmful algal blooms [6]. Estuarine surface sediments, lying at the interface between the oxidized water column or atmosphere and the deeper reduced sediment, can serve as important removal sites for inorganic nitrogen [7]. Benthic dissimilatory nitrate reduction includes three primarily anoxic processes with varying importance [8–11]: nitrate can be retained in the system as biologically available ammonium via dissimilatory nitrate/nitrite reduction to ammonium (DNRA) or lost by reduction to a gaseous product via anaerobic ammonium oxidation (anammox) or denitrification, *i*.*e*. the respiratory reduction of nitrate to either the potent greenhouse gas nitrous oxide or the harmless dinitrogen gas. Denitrification is most likely to dominate in temperate coastal areas [11]. It is proposed to be favoured over DNRA with decreasing salinity [11, 12] and lower temperatures [9, 11], and outcompetes anammox in highly variable, eutrophic estuaries [13–15].

Denitrification is performed by a wide variety of phylogenetically unrelated microorganisms. Therefore, denitrifying communities are commonly characterized using the two genes encoding cytochrome cd_1_ and copper-containing nitrite reductases (*nirS* and *nirK* respectively) as a proxy [16]. These key enzymes convert fixed nitrogen to a gaseous form, as such distinguishing dissimilatory nitrate-reducing bacteria that produce nitrite as end-product from true denitrifiers. The two forms are functionally equivalent but structurally different and respond to different environmental drivers, most of which are currently still unknown [17, 18]. Recently, elaborate genome analyses showed that both *nir* genes are not mutually exclusive in a single organism [19]. However, the functionality of these two nitrite reductases in one organism remains to be demonstrated. *NirS* denitrifiers are more widespread, whereas *nirK* denitrifiers comprise more diverse taxa [18]. Despite the importance of denitrification in estuarine systems, little is known about the diversity and distribution of the two denitrifying guilds in these ecosystems and almost no attempts are made to target both, either due to unsuccessful amplification of *nirK* or the assumption that *nirS* denitrifiers are more important *in situ* because of their numerical dominance [20].

In addition to denitrifying microorganisms, biofilm-producing microphytobenthos (MPB) present in the uppermost mm of sediments can also influence benthic nitrate reduction. MPB metabolism decouples nitrification-denitrification through (i) competition for ammonium and nitrate between MPB and bacteria [21] and (ii) pH increase via CO_2_ removal from the pore water [22]. MPB inorganic nitrogen assimilation can even exceed N consumption via denitrification by one to two orders of magnitude [21, 23], depending on the *in situ* nitrate concentrations. Diatoms, which often dominate primary production in estuarine intertidal sediments, are also known to store nitrate intracellularly up to a few 100 mM [24–26], and even use DNRA as dark survival strategy, releasing ammonium to the environment. Thus, MPB intervene in nitrogen cycling but if and how they shape the denitrifier communities *in situ* is not known.

Besides for inorganic nitrogen, other types of algal-bacterial coupling exist in these complex estuarine ecosystem [27]. Photosynthetically fixed carbon by MPB is transferred to heterotrophic bacteria within hours, resulting in a quick use of labile biofilm DOC and hydrolysed EPS [28–32], while MPB can also produce cytotoxins that can inhibit bacterial growth [33]. These algal-bacterial interactions are species-specific, both for diatoms and bacteria [30, 34, 35]. However, the effects of increased MPB biomass on bacteria in underlying sediments, including higher bacterial enzymatic activity [35] and EPS production [36] but without or with only a small increase in total bacterial cell numbers [36–40], remains ambiguous.

Based on the current knowledge, we hypothesized that higher MPB biomass (i) does not affect total bacterial abundances, (ii) negatively impacts denitrifier abundances, (iii) results in different total bacterial and denitrifier community structure, and (iv) generates differential responses of *nirK* and *nirS* denitrifier communities. To investigate these hypotheses, we sampled estuarine sediments at the Paulina polder tidal flat (Westerschelde estuary, SW Netherlands), characterized by the presence of MPB biofilms stabilizing sediment surfaces [37, 41]. The eutrophied Westerschelde estuary has a nitrogen load of 5 × 10^9^ mol N yr^–1^ [42] with nitrate being the predominant form of reactive nitrogen [43] and denitrification as the main nitrate removing process [44, 45]. Sediment samples solely differed in MPB biomass but not MPB composition. Abundance and diversity of the total bacterial community as well as both *nirS* and *nirK* denitrifying guilds were assessed using qPCR and 454 pyrosequencing. Abundant and non-abundant fractions of all three bacterial communities (*i*.*e*. all bacteria, *nirK*, *nirS*) were examined separately to also assess influences of the low-abundant fraction of the bacterial community on diversity parameters.

## Experimental procedures

### Sampling and analytical procedures

**Fig 1:**
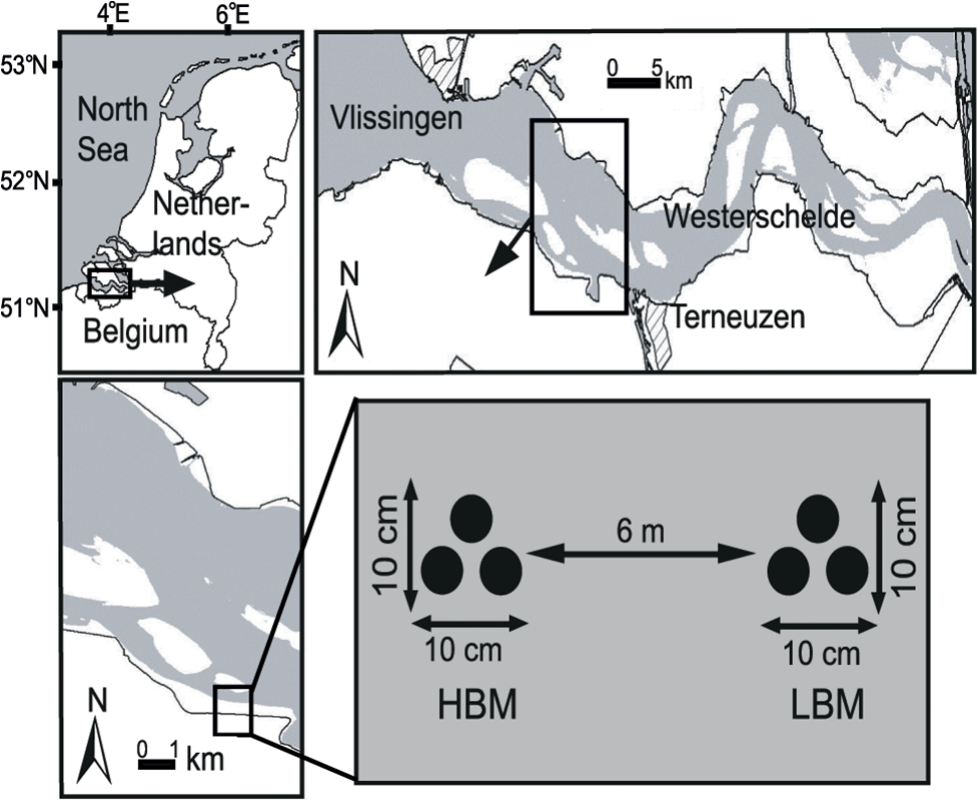
Geographical location of the Paulina tidal flat (Westerschelde estuary, SW Netherlands) and sampling design. For both estuarine sediments types (HBM and LBM) triplicate samples were taken as close as technical constraints allowed. Additional cores for measuring physico-chemical parameters were taken in immediate vicinity of the sample cores (not shown on figure).

Samples were collected in October 2011 at the Paulina polder mudflat (51° 21' 24" N, 3° 42' 51" E) in collaboration with NIOZ, which provided the necessary permit for field sampling, issued by the ‘Provincie Zeeland, The Netherlands; Directie Ruimte, Milieu en Water’. A plexiglas corer (inner Ø 3.2 cm) was used to collect triplicate samples of bacterial communities in two muddy sediments. To assess the sole effect of MPB on total bacterial and denitrifier abundance and diversity, two adjacent (± 6 m), physico-chemically similar sediments with visually different MPB biofilm development (high (HBM) or low (LBM) biomass of MPB) were sampled (MPB biofilms are visible as a brown film on the sediment; Fig 1). In both LBM and HBM sediments, the three replicates were taken as close together as technical constraints would allow, *i*.*e*. within a 10 x 10 cm square (Fig 1). The sediment cores were sealed and kept at 4°C until further processing. In the lab, the upper cm of the sediment was sampled and stored in sterile falcon tubes at -20°C until DNA extraction. At each location three further cores (each in triplicate) were taken for nutrient analyses (inner Ø 6.2 cm), determination of total organic matter (TOM) and grain size (inner Ø 3.2 cm) and determination of extracellular polymeric substances (EPS) and chlorophyll *a* (inner Ø 3.2 cm). These additional cores were taken adjacent to the cores for bacterial community structure analysis. The upper one cm of these cores was immediately frozen at -80°C (for pigment analysis) or -20°C (for all other parameters) until further analysis. The samples were analysed for NO_3_^-^/NO_2_^-^/NH_4_^+^/Si/PO_4_^-^ pore water concentrations (SAN^plus^ segmented flow analyzer, SKALAR), the total amount of organic matter (loss of mass after incineration at 500°C for 2 hours) and grain size distribution using laser diffraction (Malvern Mastersizer 2000). To assess MPB biomass in the sediment [46], chl *a* concentration was measured by HPLC analysis after pigment extraction using 10 ml 90% acetone—10% milliQ water solution [47]. Colloidal extracellular polymeric substances (cEPS) were determined by spectrophotometry using the phenol-sulfuric acid assay [48]. Furthermore, an additional single core was taken from each sediment type (inner Ø 6.2 cm, n = 1) to measure profiles of dissolved oxygen concentrations (vertical increments of 0.2 mm) and pH (increments of 1mm) in the laboratory in triplicate using Unisense microsensors (25 μm and 500 μm tip size for oxygen and pH respectively) and was used to determine the mm depth of the oxic-anoxic border and the geometric mean of the pH. Mean difference analysis using a t-test confirmed that both HBM and LBM sediments indeed only differed in parameters which could be related to MPB activity (*i*.*e*. chl *a*, EPS, phosphate, silicate), but not in other physical or chemical parameters (Table 1).

**Table 1:** Physico-chemical parameters of both estuarine sediments (HBM and LBM) (n = 3).

Parameter	HBM	LBM
pH*	6.69 ± 0.26	7.07 ± 0.19
oxic-anoxic interface (mm depth)	5.25 ± 0.99	6.51 ± 0.64
TOM ^a^ (%)	5.73 ± 0.67	4.65 ± 0.42
Chl *a* * (μg/g dw)	28.61 ± 1.61	13.26 ± 3.48
EPS ^b^ ^,*^ (mg/mg dry sediment)	0.0004 ± 0.00015	0.0002 ± 0.00003
% mud ^c^	51.26 ± 9.92	45.94 ± 5.08
NH_4_^+^ ^d^ (μg/l)	3 632.58 ± 434.83	4 445.93 ± 2870.13
NO_3_^-^ ^d^ (μg/l)	1 376.85 ± 281.89	761.33 ± 351.88
NO_2_^-^ ^d^ (μg/l)	10.17 ± 2.31	16.24 ± 4.51
PO_4_^3-^ ^d^ ^,*^ (μg/l)	1 058.94 ± 66.53	1 918.8 ± 1276.08
Si ^d^ ^,*^ (μg/l)	2 125.45 ± 622.9	4 356.73 ± 206.36

Significant differences (*) between both sediments (t-test, p ≤ 0.05) were detected using a t-test (p ≤ 0.05).; for PO_4_^3-^, a non-parametric test (Mann-Whitney U) was performed. Reliability of significance testing was checked using the Levene’s p-value (>0.05). Millimeter depth till oxic-anoxic border and pH were excluded from statistical testing as no biological replicates were taken for these parameters.

a. TOM, total organic matter.

b. Extracellular polymeric substances.

c. Percentage mud (particle size < 63μm) determined using the Wenthworth grain size chart

d. Pore water concentrations.

### DNA extraction

DNA was extracted in triplicate from each HBM and LBM biological replicate (only top 0–1cm) separately to account for both technical and biological variation using a slight modification of Boon et al. [49]. In brief, one gram of sediment, 750 μl 1x TE buffer (1 mM EDTA, 10 mM Tris-HCL, pH8) and 0.5 g of glass beads (Ø 0.1 mm) were added to a 2 ml Safe lock tube (Eppendorf). The mixture was vigorously shaken three times for 90 s using a bead beater (Mixer Mill MM200, Retsch) at a frequency of 30 Hz. Then, 150 μl of lyzosyme (6 mg /150 μl 1x TE buffer) was added and gently mixed for 5 minutes at room temperature. Subsequently, 40 μl of 20% SDS was added, and samples were again slowly mixed for 5 min at room temperature, with subsequent addition of 250 μl 8M ammonium acetate. The supernatant was collected after centrifugation at 7 000 rpm for 15 min at 4°C. A chloroform-isoamylalcohol (24:1) purification was performed, followed by centrifugation at 7 000 rpm for 15 min at 4°C. The aqueous phase was transferred to a new Safe lock tube, and 0.8 volume of isopropanol was added. The precipitation was performed for 1 h at -20°C. Finally, the pellet was obtained by centrifugation at 12 000 rpm for 25 min, washed in 70% ethanol (5 min at 12 000 rpm) and resolved in 50 μl of 1x TE buffer. DNA yields were determined using a Nanodrop 2000 spectrophotometer (Thermo, Scientific) and the quality was checked by gel electrophoresis. Finally, DNA triplicates of a single biological replicate were mixed together for further analysis.

### Barcoded amplicon sequencing of *nirK*, *nirS* and 16S rRNA genes

Amplicon libraries were prepared using a two-step PCR procedure as recommended by Berry et al. [50] for the 16S rRNA, *nirK* and *nirS* genes. Six different multiplex identifiers (MIDs) were used to identify the different replicates per sediment type (S1 Table). Targeting the V3-V1 region of the 16S rRNA, 27 cycles of amplification were performed with the F19-38 (CTGGCTCAGGAYGAACGCTG [51]) / 518R (ATTACCGCGGCTGCTG [52]) primer set. Sequencing starting from the V3 region was selected as it was previously shown to result in good taxonomic assignment [53, 54]. Furthermore, the V1-V3 region is often targeted in sequencing projects and therefore well represented in publically available databases. In a second step, 2 μl of the PCR products of the first reaction was amplified in a 5 cycle PCR with the barcoded PCR primers including the 10 basepair-MID, a sequencing key and the sequencing primer (S1 Table). Each PCR reaction was performed in a 25 μl volume using 1.25 U FastStart High Fidelity Enzyme Blend (Roche), 0.1 μM primers, 0.2 mM dNTP mix and 0.1 mg bovine serum albumin (BSA, only used on the first round of PCR) and milliQ water. All reactions were performed in triplicate to take into account technical variation and were pooled before determination of PCR product quantity and quality. The thermal program consisted of an initial denaturation step of 95°C for 3 min, a cycling program of 94°C for 30 s, 57°C for 45 s, and 72°C for 120 s. Before sequencing, PCR amplicons were purified using the MinElute purification kit (Qiagen) and quantified with a fluorescent stain-based kit (Quant-iT Pico Green, Invitrogen). The quality of the PCR product was assessed on a Bioanalyzer 2100 using a high sensitivity DNA chip (Aligent). Amplicon libraries for the *nirK* and *nirS* genes using the primer pairs F1aCu (ATCATGGTSCTGCCGCG)—R3Cu (GCCTCGATCAGRTTGTGGTT) [55] and Cd1aF (GTSAACGTSAAGGARACSGG)—R3cd (GARTTCGGRTGSGTCTTGA) [56, 57] were prepared in a similar way as described above except that the number of cycles in the first PCR round was increased to 35 cycles. All amplicons were sequenced unidirectionally, starting from the forward primer for *nirK* and *nirS* and the reverse primer for 16S rRNA gene. Sequencing was performed on a GS FLX Titanium at the NXTGNT sequencing facility of Ghent University.

### Sequence analyses

All sequence data were screened and de-multiplexed using default setting of MOTHUR v.1.30.1 [58]. Sequences containing homopolymers of more than 8 nucleotides, mismatches to the barcode (>1) and primers (>2) and sequences shorter than 200 bp were discarded. Subsequently, a MOTHUR-implemented version of pyronoise was used to further denoise the data. Chimera sequences were removed using the Uchime algorithm [59] and, in case of the 16S rRNA gene, potential chloroplast and mitochondrial sequences were also removed. The 16S rRNA gene sequences were aligned using the SILVA reference alignment (release 102) and binned into operational taxonomic units (OTUs) at a 97% gene sequence identity threshold. Sequences were classified using a MOTHUR formatted version of the RDP training set (v.9).

Prior to alignment, the *nirK*/*nirS* gene sequence data were checked for the presence of specific functional/conserved regions and screened using the HMM FRAME algorithm [60] included in the FunFrame pipeline [61] to detect and correct frameshift errors. The obtained HMM alignment scores were used for further quality filtering. The cytochrome d1 HMM from Pfam (accession PF02239.10) was used for the *nirS* gene sequence dataset and sequences with a HMM score ≥ 107 were retained. The *nirK* primers targeted a region that overlaps with two domains, plastocyanin-like 1 and plastocyanin-like 2, of the *nirK* gene and a HMM was designed covering the primer target region based on sequences obtained from the Fungene database using the HMMER3 program (Hmmer.org). *NirK* gene sequences with a HMM score ≥ 48 were retained and for both genes pairwise alignment was performed with the remaining sequences using MOTHUR v.1.30.1 [58]. Cut-offs for binning *nirS* and *nirK* gene sequences into OTUs were determined experimentally with the focus on only retaining functional diversity, *i*.*e*. binning identical amino acid sequences. A range of threshold distances (5–20%) was tested for both the *nirK* and *nirS* gene sequences using MOTHUR v.1.30.1 [58]. Subsequently, all OTU representative gene sequences per threshold distance were translated *in silico* and pairwise distance matrices of amino acid sequences of all OTU representatives were determined using MEGA 5.10 [62]. At a cut-off of 82% gene sequence identity, all pairwise distances of *in silico* translated AA sequences of all *nirK* OTU representatives were > 0, indicating that the sequences of all OTU representatives had a unique AA sequence (~functional diversity). In case of the *nirS* gene, a similarly obtained cut-off of 80% sequence identity was used for OTU binning. Maximum likelihood phylogenies for both genes were calculated using RAxML 7.4.2 [63, 64]. Protein BLAST searches with the OTU representatives were performed to determine the closest relatives using three different NCBI databases: a non-redundant protein database with/without uncultured/environmental sequences and the whole genome database. If possible, depending on alignable length and e-value, the first five hits were used for further analysis. Node confidence was determined using 1 000 bootstrap replicates.

### Quantification of *nirK*, *nirS* and 16S rRNA genes

Quantitative real-time PCR (qPCR) of *nirK*, *nirS* and 16S rRNA genes was carried out using a Lightcycler 480 II (Roche, Applied Science). Standard curves were prepared from serial dilutions of linearized plasmid with the *nirK* gene from *Alcaligenes faecalis* LMG 1229^T^, *nirS* gene from *Paracoccus* sp. R-24615 and 16S rRNA gene from *Flavobacterium swingsii* LMG 25510, containing between 10^9^ to 10^1^ target gene copies calculated directly from the concentration of the extracted plasmid. DNA concentrations were determined using the Nanodrop 2000 spectrophotometer (Thermo, Scientific). The qPCR assays were carried out in a 20 μl reaction volume composed of SensiMix SYBR No-ROX (Bioline GmbH, Luckenwalde, Germany), 0.4 μM of each primer, 2.5 μl of template DNA (10 ng/μl), 0.1 mg BSA (not used in the *nirS* assay) and sterilized milliQ water. The same primers as for pyrosequencing were used and the thermal protocol can be found in S2 Table. All reactions per sample were performed in triplicate. Agarose gel electrophoresis, melting curve analysis, cloning and sequencing of the obtained amplicons indicated that the amplification was specific. PCR inhibition was determined by spiking sediment DNA with a known amount of standard DNA and corrected for according to Zaprasis et al. [65].

### Statistical analysis

Chao1 estimations, Inverse Simpson diversity indices, rarefaction curves, Venn diagrams, community membership (Jaccard dissimilarity index) and structure (Bray Curtis dissimilarity index) were calculated using MOTHUR v.1.30.1 [58]. Mean differences between HBM and LBM sediment samples were tested using a t-test. Further, correlations between physico-chemical parameters and abundance data from the six collected samples were analysed using product moment correlations (Statistica 5.0, Statsoft 1984–1995). Homogeneities of variances were checked using the Levene’s test (p > 0.05). For PO_4_^3-^, non-parametric Mann-Whitney U tests and Spearman’s rank correlations were used since the necessary assumptions for heterogeneity of variances and normal distributions were not met. Permutational analyses of variance (Permanova) were conducted using the Permanova add-on software for Primer v6 [66].

### Nucleotide sequence accession numbers

Complete amplicon libraries of 16S rRNA, *nirK* and *nirS* gene sequences derived from barcoded amplicon sequencing were deposited in the Genbank SRA database under study accession number SRP035903.

## Results

### Sediment sampling and physico-chemical analysis

Two adjacent (± 6 m) sediments with visually different MPB biofilm development were sampled in triplicate. MPB communities in all HBM and LBM replicates were dominated mainly by *Navicula* spp. (*Navicula arenaria* var. *rostellata*, *N*. *phyllepta* and *N*. *gregaria*) and contained to a lesser degree also *Gyrosigma fasciola*, *Amphora copulata* and an unknown *Nitzschia* species. No significant differences could be observed between the HBM and LBM sediments in TOM, % mud content and pore water concentrations of NO_3_^-^, NO_2_^-^ and NH_4_^+^ (Table 1). So, the only detected differences between HBM and LBM sediments were specifically related to the presence and activity of MPB: chlorophyll *a*, a proxy for MPB biomass, and EPS were significantly higher in the HBM sediments, while PO_4_^3-^ and Si were significantly lower (p < 0.05, Table 1).

### Total bacterial community diversity, estimated richness and structure

The rarefaction curves (S2 Fig) and the ratio observed:estimated 16S rRNA OTU richness for samples HBM1, HBM3, LBM1 and LBM2 (0.72–0.84, Table 2) indicated that the current sampling effort was almost sufficient to capture total bacterial diversity, but showed clear differences between HBM and LBM samples (0.72–0.76 vs. 0.84–0.85; Table 2). The 16S rRNA gene rarefaction curves of a replicate per sample type (HBM2 and LBM3) flattened very quickly in comparison with the other replicates (S2 Fig). As no such discrepancies were observed when using the same DNA material for *nir* gene sequencing and qPCR (see below), we suspect that the limited numbers of OTUs observed for HBM2 and LBM3 resulted from inconsistent emulsion PCR and therefore excluded both samples from further analyses. After removal of HBM2 and LBM3 sequences from the data set, a total number of 149 946 sequences were binned into 2 482 OTUs across all samples at a cut-off of 0.03. In total, 2.69% of all sequences were determined as chimera or chloroplast and removed from the data set. Both the observed and estimated OTU richness in LBM samples were significantly (p <0.05) higher than in HBM samples, with the majority of OTUs being non-abundant (i.e. relative abundance < 1%; Table 2). Singletons (832 OTUs) and doubletons (485 OTUs) together accounted for approximately half of the total number of OTUs. Given the higher OTU richness in the LBM sediments, % coverage was also significantly lower than in the HBM sediments (p < 0.05, Table 2). Despite this higher richness in LBM samples, the inverse Simpson diversity index did not significantly differ between the HBM and LBM sediments (Table 2).

**Table 2:** Overview of 16S rRNA (n = 2), *nirK* (n = 3) and *nirS* (n = 3) gene sequences derived from HBM and LBM estuarine sediments.

				Richness			Diversity
Target	Sample	# sequences	Library coverage^a^ (%)	# OTUs observed^b^	# OTUs estimated^c^	Observed/estimated ratio	Inverse Simpson ^d^
**16S rRNA**	**HBM1**	31 415	99.57	779 (A:14-NA:765)	1021 (927–1172)	0.76	26.70 (26.07–27.36)
	**HBM3**	37 298	99.5	821 (A:13-NA:808)	1138 (1008–1347)	0.72	34.83 (34.06–35.63)
	**LBM1**	42 512	98.86	1235 (A:16-NA:1219)	1468 (1385–1578)	0.84	24.71 (24.21–25.23)
	**LBM2**	38 721	99.02	1232 (A:15-NA:1217)	1454 (1379–1554)	0.85	33.76 (33.04–34.52)
***nirK***	**HBM1**	13 510	99.88	59 (A: 11-NA:48)	63 (57–89)	0.94	4.30 (4.20–4.42)
	**HBM2**	13 602	99.9	52 (A:7-NA:45)	61 (57–80)	0.85	4.67 (4.55–4.79)
	**HBM3**	9 028	99.84	59 (A:13-NA:46)	65 (56–98)	0.91	3.41 (3.32–3.51)
	**LBM1**	11 701	99.87	56 (A:5-NA:51)	68 (59–101)	0.87	4.05 (3.93–4.18)
	**LBM2**	9 779	99.82	54 (A:8-NA:46)	68 (59–98)	0.82	2.11 (2.06–2.16)
	**LBM3**	10 802	99.89	59 (A:10-NA:49)	57 (54–75)	0.95	3.50 (3.42–3.59)
***nirS***	**HBM1**	6 365	99.64	58 (A:7-NA:51)	71 (59–108)	0.82	3.56 (3.48–3.65)
	**HBM2**	16 427	99.76	61 (A:8-NA:53)	59 (50–91)	1.03	3.44 (3.38–3.50)
	**HBM3**	4 948	99.71	47 (A:7-NA:40)	60 (51–93)	0.78	1.98 (1.92–2.04)
	**LBM1**	6 948	99.69	57 (A:8-NA:49)	65 (56–97)	0.88	3.27 (3.20–3.34)
	**LBM2**	6 684	99.68	57 (A:7-NA:50)	66 (56–97)	0.86	2.31 (2.25–2.38)
	**LBM3**	17 548	99.68	72 (A:7-NA:65)	70 (59–103)	1.03	3.62 (3.56–3.68)

a. Good’s coverage estimates sampling completeness and calculates the probability that a randomly selected amplicon sequence from a sample has already been sequenced.

b. A, abundant OTUs (> 1% relative abundance); NA, non-abundant (< 1% relative abundance).

c. Chao1 richness with upper and lower 95% confidence intervals.

d. Inverse Simpson diversity index with upper and lower 95% confidence intervals.

**Fig 2:**
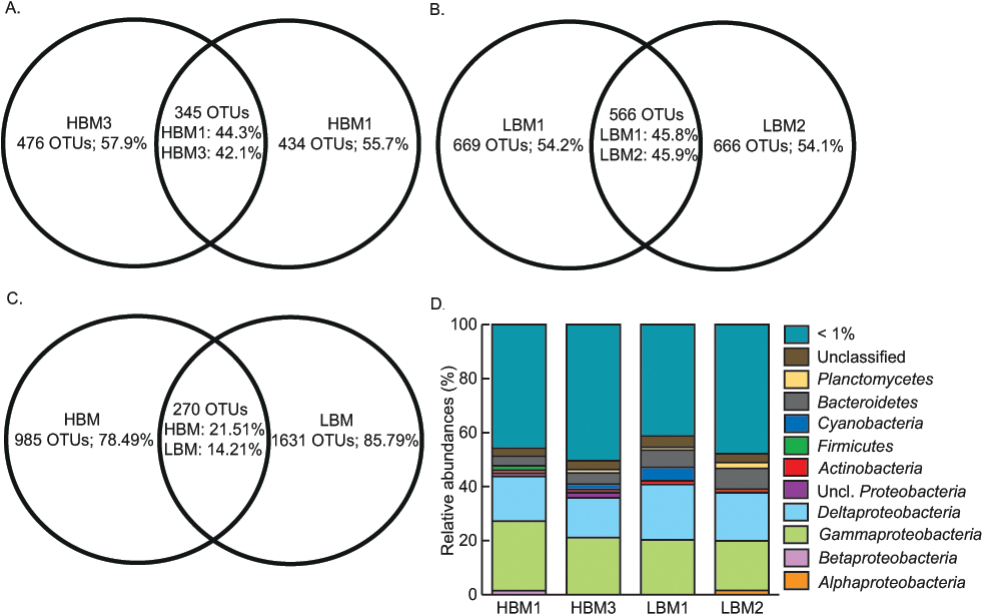
Distribution of 16S rRNA gene OTUs. A-C, Venn diagrams representing the number of observed OTUs for the 16S rRNA gene. Comparisons are shown between (A) HBM replicates, (B) LBM replicates, (C) HBM (n = 2) and LBM (n = 2) samples. The number and percentage of unique and shared OTUs are given. D, The relative abundance of abundant 16S rRNA derived OTUs, grouped per phylum, from HBM (n = 2) and LBM (n = 2) sediment samples. Sequences were assigned to OTUs using sequence dissimilarity treshold of 3%. All OTUs with a relative abundance below 1% were grouped. Uncl. stands for unclassified.

Replicates of each sediment type shared only about half of their total number of OTUs (Fig 2A and 2B); this variation among replicates could be attributed to a high dissimilarity in non-abundant OTUs. Both sample types showed clear differences in community members, although not significant and again mostly among non-abundant OTUs (Table 3), with 78.5% of HBM OTUs and 85.8% of LBM OTUs being unique (Fig 2C). Nevertheless, community structures (Bray-Curtis dissimilarity index, which takes into account community membership as well as OTU relative abundances) did not show significant differences at any level (total community, abundant and non-abundant fraction) between both sample types (Table 3).

**Table 3:** Dissimilarity in community membership (Jaccard) and structure (Bray-Curtis) between both estuarine sediment types (HBM and LBM) based on 16S rRNA, *nirK* and *nirS* genes.

		Jaccard	Bray-Curtis
**All OTUs**	**16S rRNA**	0.732897	0.299214
	***nirK***	0.153846	0.185174
	***nirS***	0.255556	0.065105
**Abundant OTUs**	**16S rRNA**	0.357143	0.260035
	***nirK***	0.411765	0.181191
	***nirS***	0.222222	0.0589
**Non-abundant OTUs**	**16S rRNA**	0.735594	0.402036
	***nirK***	0.213333	0.430228
	***nirS***	0.277108	0.48913

Three different levels were assessed: total community (all), abundant and non-abundant fraction. An OTU was defined as abundant when its relative abundance was larger than 1%. Permanova analyses were performed to determine significant differences in community structure and membership.

### Taxonomic diversity in HBM and LBM samples

**Fig 3:**
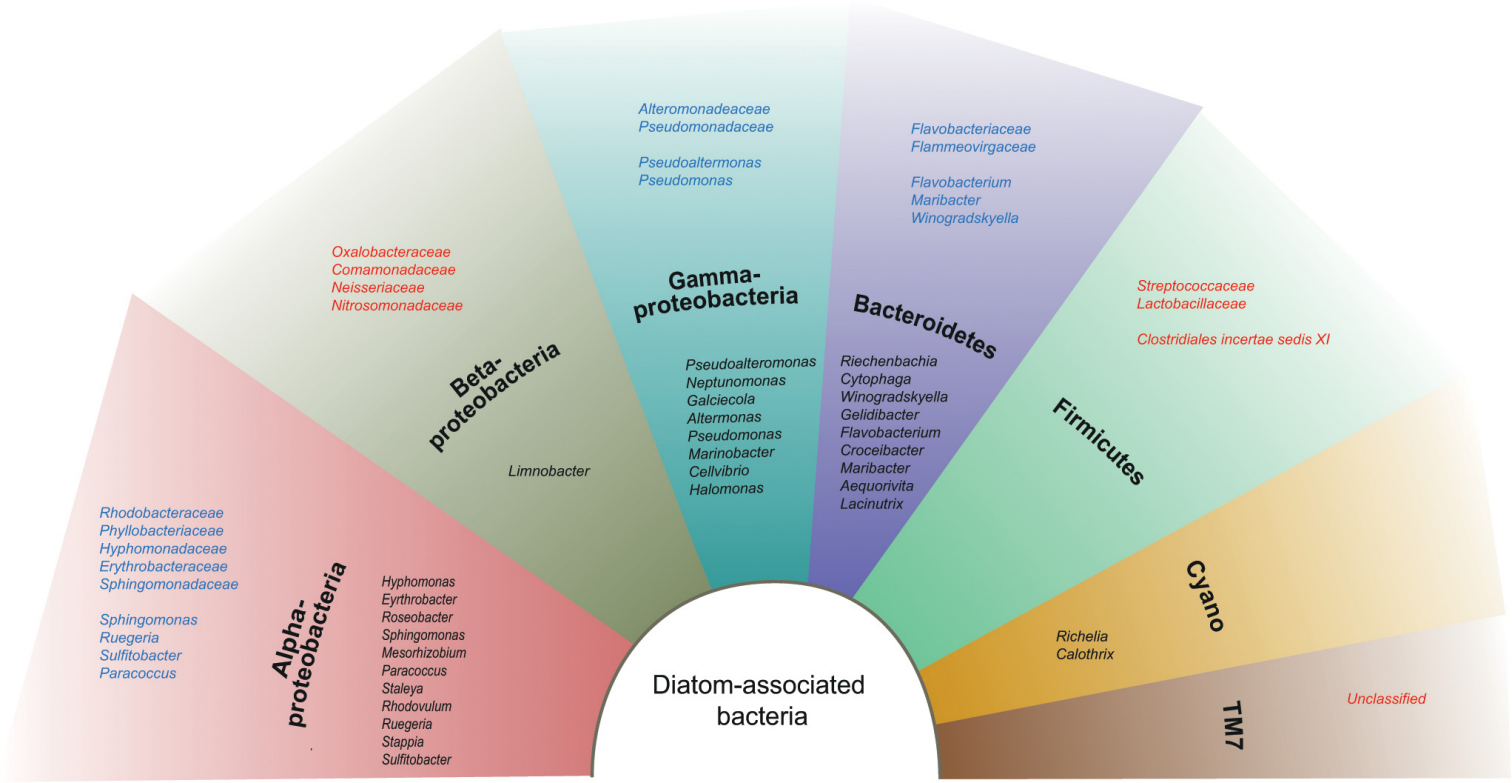
Overview of diatom-associated bacteria found in different phyla. The inner tier represents diatom-associated bacterial taxa reported by Amin et al. (35). The outer tier depicts diatom-associated bacterial taxa found in our study, either previously reported (blue) or representing potentially new diatom- bacteria associations (red). The highest taxonomic identification of these taxa is shown. Diatom-bacteria associations were identified based on the difference in relative abundances of specific taxa (*i*.*e*. number of sequences per taxon) between HBM and LBM sediment samples.

The estuarine sediments harboured bacteria belonging to a broad range of known phyla, with approximately one third of the OTUs remaining unclassified (30.58% or 759 OTUs, Table 4). The majority of known OTUs belonged to the *Proteobacteria* (representing one third of all OTUs), *Bacteroidetes*, *Planctomycetes*, *Actinobacteria*, *Acidobacteria* and *Verrucomicrobia*. Candidate phyla OD1, OP11, SR1, TM7 and WS 3 were represented by only a few OTUs (Table 4). Twenty-one OTUs were found to have a relative abundance of more than 1% in at least one of the replicates of both sediment types. These abundant OTUs belonged to *Alpha*- (1 OTU), Beta- (1 OTU), *Gamma*- (6 OTUs), *Delta*- (4 OTUs) and unclassified *Proteobacteria* (1 OTU), *Actinobacteria* (1 OTU), *Firmicutes* (1 OTU), *Cyanobacteria* (1 OTU), *Bacteroidetes* (2 OTUs), *Planctomycetes* (1 OTU) and two OTUs with no taxonomic identification (Fig 2D). The phyla *Armatimonadetes*, *Chlorobi*, *Spirochaetes* and *Tenericutes* appeared to be unique to LBM samples, although they were only represented by one to five OTUs. A significant difference in the number of OTUs between HBM and LBM samples was found for five phyla, namely for *Actinobacteria*, *Bacteroidetes*, *Proteobacteria* (more specifically *Alpha-*, *Gamma-*, *Delta-* and unclassified), *Spirochaetes*, and *Verrucomicrobia*, as well as the unclassified fraction (p < 0.05) (Table 4). Eighteen bacterial taxa (families or genera within *Alpha*-, *Gammaproteobacteria or Bacteroidetes)* were detected that were previously described to harbour diatom-associated bacteria [34] (Fig 3).

**Table 4:** Detection and distribution of bacterial phyla (or subdivisions of *Proteobacteria*).

Phylum		Total	HBM	LBM
		% (# OTUs)	% (# OTUs)	% (# OTUs)
*Acidobacteria*		3.63 (90)	3.59 (45)	4.10 (78)
*Actinobacteria*		4.67 (116)	6.06 (76)*	4.73 (90)*
*Armatimonadetes*		0.04 (1)	0 (0)	0.05 (1)
*Bacteroidetes*		9.31 (231)	8.45 (106)*	9.21 (175)*
*Chlamydiae*		0.16 (4)	0.16 (2)	0.16 (3)
*Chlorobi*		0.08 (2)	0 (0)	0.11 (2)
*Chloroflexi*		1.69 (42)	1.83 (23)	1.68 (32)
*Cyanobacteria*		0.73 (18)	0.80 (10)	0.58 (11)
*Deinococcus-Thermus*		0.04 (1)	0.08 (1)	0 (0)
*Firmicutes*		2.98 (74)	3.19 (40)	2.74 (52)
*Fusobacteria*		0.08 (2)	0.08 (1)	0.11 (2)
OD1		1.49 (37)	1.91 (24)	1.10 (21)
OP11		0.32 (8)	0.32 (4)	0.26 (5)
*Planctomycetes*		6.21 (154)	4.06 (51)	7.21 (137)
*Proteobacteria*	*Alphaproteobacteria*	5.04 (125)	5.82 (73)*	4.94 (94)*
	*Betaproteobacteria*	1.09 (27)	1.59 (20)	0.95 (18)
	*Gammaproteobacteria*	14.67 (364)	15.62 (196)*	14.52 (276)*
	*Deltaproteobacteria*	6.93 (172)	6.69 (84)*	7.63 (145)*
	*Zetaproteobacteria*	0.04 (1)	0.08 (1)	0 (0)
	*Epsilonproteobacteria*	0.08 (2)	0.08 (1)	0.11 (2)
	Unclassified	2.54 (63)	2.23 (28)*	2.47 (47)*
*Spirochaetes*		0.20 (5)	0 (0)*	0.26 (5)*
SR1		0.16 (4)	0.08 (1)	0.16 (3)
*Synergistetes*		0.12 (3)	0.16 (2)	0.05 (1)
*Tenericutes*		0.04 (1)	0 (0)	0.05 (1)
TM7		1.09 (27)	1.59 (20)	1.00 (19)
*Verrucomicrobia*		5.48 (136)	6.14 (77)*	5.89 (112)*
WS3		0.52 (13)	0.64 (8)	0.53 (10)
Unclassified		30.58 (759)	28.76 (361)*	29.41 (559)*

Percentages OTU per phylum are given for both sediment types (Total) and for each sediment type separately (HBM and LBM), exact numbers are given between brackets. Phyla containing previously described diatom-associated bacteria are underlined. Significant differences (*) in the number of OTUs between HBM and LBM samples were detected using a t-test (p ≤ 0.05). Reliability of significance testing was checked using the Levene’s p-value (>0.05).

### Denitrifying guild diversity, estimated richness and structure

In total, 68 422 quality filtered *nirK* gene sequences, including 4 693 unique sequences, were assigned to 78 OTUs, with six singleton OTUs as and two doubletons. Comparable numbers of OTUs were found in HBM and LBM samples (Table 2). Rarefaction curves (S3A Fig) and the ratio observed:estimated OTU richness (0.82–0.94, Table 2) indicated that the sequencing effort was almost sufficient to cover the whole *nirK* diversity detectable with the applied primer set in both sediment types. HBM and LBM samples had similar richness and diversity values (Table 2), with both containing nine abundant OTUs and being dominated by OTUs 1 and 2 (S4A Fig). Although three biological replicates were taken from each sediment type, differences in relative abundances of abundant OTUs between replicates within sample types were as large as between sample types (S4A Fig), while non-abundant OTUs were very similar.

Looking at the *in silico* translated amino acid (AA) sequences of *nirK*, the majority of all OTU representatives clustered together with *Alphaproteobacteria*—spread over four clusters—and *Betaproteobacteria* (S5 Fig). One distinct cluster (supported by a high bootstrap value of 100%) did not contain known cultivated representatives. Sequences derived from *Bacteriodetes* and *Firmicutes* were included but proved unrelated to our OTU representatives. Most OTU representatives clustered together with sequences obtained from marine environments, with the exception of two OTUs (OTU 11 and 66) closely related to sequences from soil.

In total, 58 920 quality filtered *nirS* gene sequences, with 5 391 unique sequences, were assigned to 90 OTUs, with twelve singleton OTUs and eighteen doubletons. Comparable numbers of OTUs were found in HBM and LBM samples (Table 2). As for *nirK*, rarefaction curves (S3B Fig) and the ratio observed:estimated OTU richness (0.78–1.03, Table 2) indicated that the current sequencing effort was nearly sufficient to completely catalogue the *nirS* diversity detectable with the applied primer set in both sample types. HBM and LBM did not significantly differ in *nirS* gene richness and diversity. OTUs 1–3 dominated in both sediments, and nine OTUs were abundant (S4B Fig). In contrast to *nirK*, replicates of a single sediment type differed less in relative abundances of abundant OTUs (S4 Fig).

Looking at the *in silico* translated amino acid (AA) sequences of *nirS*, most OTU representatives clustered together with *Alpha-*, *Beta* and also *Gammaproteobacteria* (S6 Fig). Multiple clusters without cultured representatives were found, albeit not always supported by high bootstrap values. Most of the OTU representatives clustered together with sequences obtained from marine environments; eight OTUs resembled sequences derived from soil environments or activated sludge.

**Fig 4:**
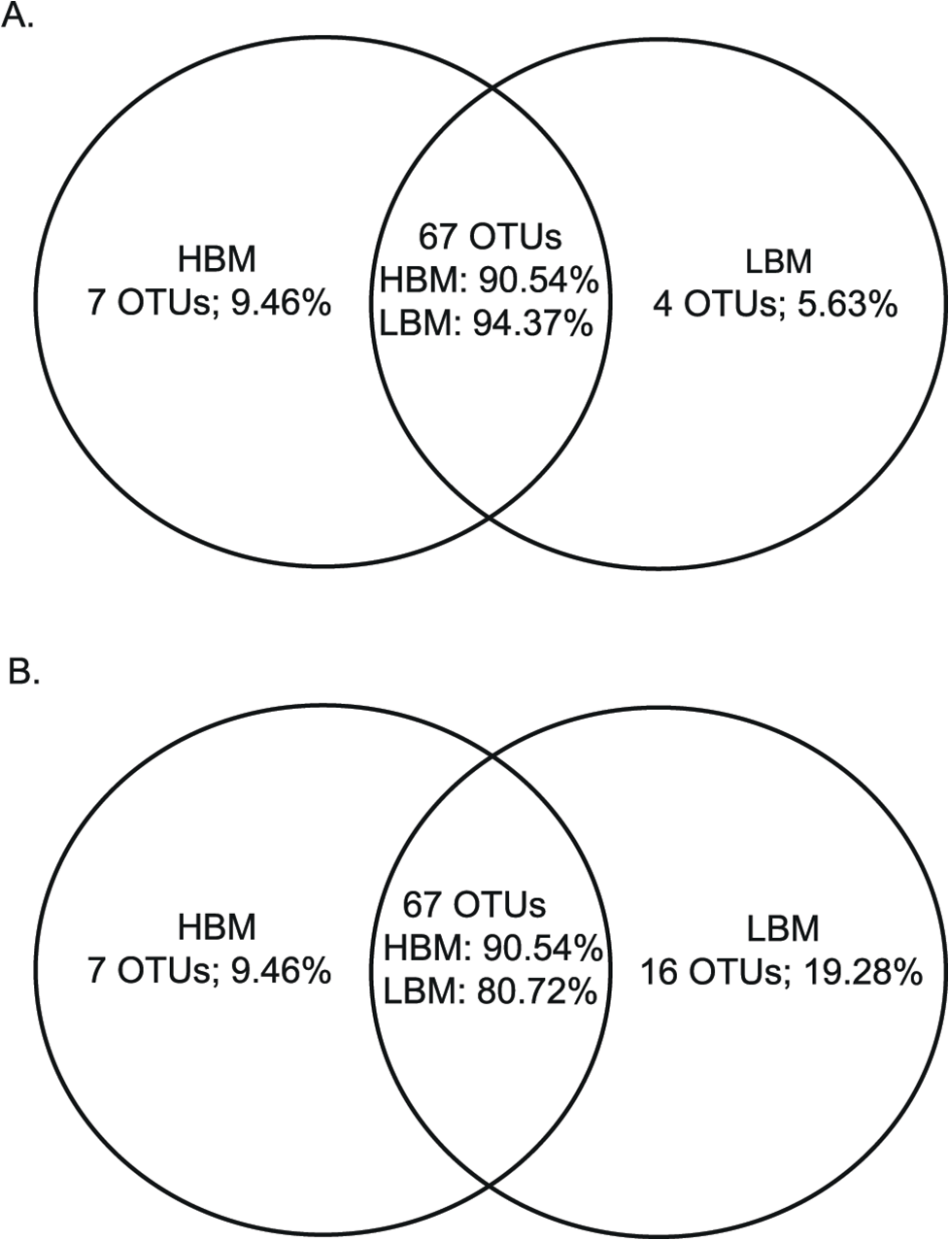
Venn diagrams representing number of observed OTUs for the *nirK* (A) and *nirS* (B) genes. Comparison is shown between HBM and LBM samples (n = 3) for both genes. The number and percentage of unique and shared OTUs are given.

*NirK* and *nirS* community membership and structure were very similar between HBM and LBM samples (Table 3), with only a few unique OTU’s for each sediment type (Fig 4A and 4B). Similar trends were found when considering the abundant and non-abundant community levels separately (Table 3). However, it is interesting to note that OTUs 12 and 13 were exclusively present in the *nirK* abundant HBM community fraction (S4 Fig).

### Quantification of the denitrifying guild abundance relative to total bacterial abundance

The abundances of both *nirK* and *nirS* denitrifiers and the total bacterial communities of HBM and LBM samples were determined via qPCR (Table 5). The PCR efficiency of the *nirS* assay (62.9%) was lower than for the other genes, probably due to the exclusion of BSA in this assay because of negative effects on the melting curves. Inhibition factors per gene type were experimentally determined and gene copy numbers were corrected accordingly. The level of inhibition for both *nir* genes was similar, but much lower than for the 16S rRNA gene. Total bacterial abundances ranged from 8.25 x 10^5^ to 7.98 x 10^7^ copies/g sediment. Final copy numbers of *nirK* and *nirS* ranged from 1.48 x 10^3^ to 6.09 x 10^4^ copies/g sediment and 3.66 x 10^4^ to 3.35 x 10^5^ copies/g sediment respectively (Table 5).

**Table 5:** Absolute and relative quantification of 16S rRNA, *nirK* and *nirS* genes in both estuarine sediment types (HBM and LBM).

Sample	Absolute quantification			Relative quantification	
	16S rRNA^a^ gene	*nirK* ^*b*^ gene	*nirS* ^*c*^ gene	ratio	ratio
	copies / g sediment	copies / g sediment	copies / g sediment	*nirK*/16S rRNA	*nirS*/16S rRNA
**HBM1**	(6.68 ± 0.85) x 10^7^	(3.39 ± 0.38) x 10^4^	(2.93 ± 0.16) x 10^5^	0.0005	0.0044
**HBM2**	(3.75 ± 0.13) x 10^7^	(5.40 ± 0.72) x 10^4^	(2.53 ± 0.18) x 10^5^	0.0014	0.0068
**HBM3**	(7.98 ± 0.48) x 10^7^	(6.09 ± 0.70) x 10^4^	(3.35 ± 0.26) x 10^5^	0.0008	0.0042
**LBM1**	(5.56 ± 0.64) x 10^6^	(1.48 ± 0.15) x 10^3^	(3.66 ± 0.60) x 10^4^	0.0003	0.0066
**LBM2**	(5.19 ± 0.40) x 10^6^	(8.61 ± 0.36) x 10^3^	(6.00 ± 0.55) x 10^4^	0.0017	0.0116
**LBM3**	(8.25 ± 0.24) x 10^5^	(2.72 ± 0.37) x 10^3^	(6.23 ± 0.14) x 10^4^	0.0033	0.0755

Analyses was performed in triplicate for each biological replicate (n = 3). Gene copy numbers given were corrected for PCR inhibition.

a. Detection limit, 10^1^ copies; PCR efficiency, 90.4%; standard curve R^2^, 0.99; inhibition factor range: 0.07–0.10.

b. Detection limit, 10^1^ copies; PCR efficiency, 87%; standard curve R^2^, 0.99; inhibition factor range: 0.36–0.62.

c. Detection limit, 10^1^ copies; PCR efficiency, 62.9%; standard curve R^2^, 0.99; inhibition factor range: 0.31–0.55.

Significant differences in the abundance of 16S rRNA, *nirK* and *nirS* genes were detected between HBM and LBM samples (p < 0.05), with the abundances of all three genes being a log unit higher in HBM samples. *NirS* genes were also consistently approximately a log unit more abundant than *nirK* genes (Table 5). For all three genes, a strong positive correlation was found with chl *a* and EPS whereas a significantly negative correlation was found for Si. Both are in agreement with the higher abundances of the all three genes in HBM sediments (p < 0.05, S3 Table). Furthermore, a positive correlation was found between 16S rRNA abundances and concentration NO_3_^-^ and *nirK* abundances and % TOM (p < 0.05, S3 Table).

## Discussion

In intertidal sediments of the Westerschelde estuary, a two-fold increase in biomass (*i*.*e*. chl *a*) of otherwise compositionally identical MPB communities coincided with a disproportionate (ten-fold) increase in both total bacterial and denitrifying community abundances in underlying sediments (Table 5). This contrasts with previous studies that reported correlations between MPB biomass and bacterial abundances with a more moderate quantitative influence (two to three-fold), but, unlike here, these studies did not exclude variation of other environmental factors non-related to MPB (e.g. salinity, temperature, seasonality) [38–40]. Unexpectedly, this two-fold increase in MPB biomass also corresponded with a ten-fold increase in denitrifier abundance. Potential competition with diatoms for nitrate appeared negligible at the NO_3_^-^ concentration in the investigated sediments (appr. 1 mg/l, *i*.*e*. 10x higher than in sediments with comparable MPB biomass with competition [21, 23]). Thus, microphytobenthos was no specific determinant of denitrifying guilds but rather influenced the bacterial population more generally. This agrees with the facultative nature of the denitrification capacity, and the assumption that the population distribution of denitrifiers is determined by their general ability to compete for natural carbon substrates under aerobic conditions [67]. Unfortunately, our results could not ascertain whether the MPB directly affected the bacterial abundance or whether (identical) non-measured variables influenced both MPB and bacteria simultaneously. Estuarine MPB biofilms can be extremely variable in time (seasonality) and space (from cm to km), with complex interactions of biotic and abiotic parameters responsible for their patchiness [68]. Tidal height, sediment type and hydrodynamism [69, 70], and intraspecies competition [71, 72] are unlikely causes as both samples, from the same sediment type, were taken at the same tidal height and both biofilms contained the same MPB species. However, grazing of MPB, alone or with bacteria, by higher organisms such as grazing benthic deposit feeding invertebrates like harpacticoid copepods [73], (bacterivorous) nematodes [74, 75], polychaetes and bivalves [76] is a plausible factor.

Besides with chl *a*, total bacterial abundances correlated with other parameters related to MPB activity, namely EPS, produced by both diatoms and bacteria [37] and a potential nutrient source for bacteria and diatoms [29, 31, 41], and Si, a major limiting nutrient for diatoms and hence an important factor controlling primary production [77]. Taxon-specific effects of diatom-derived EPS (*i*.*e*. a shift to algal organic matter degrading taxa) could explain the significant decreases in number of OTUs in *Actinobacteria*, *Bacteroidetes*, *Proteobacteria*, *Spirochaetes*, and *Verrucomicrobia* (Table 4) in HBM compared to LBM as well as the big, albeit not significant, difference in community membership, specifically for non-abundant taxa (Table 3). The same effects could also be the result of potential bactericidal effects of MPB, not measured in this study (e.g. by polyunsaturated aldehydes (PUAs) [33]). In addition to a shift in number of OTUs, diatom-derived EPS (and PUA) might also explain the increase in total bacterial abundances in HBM samples, as specialist EPS-degrading bacteria are specifically favoured by these additional nutrient sources [27, 29, 78]. Using the relative abundances of specific taxa (*i*.*e*. number of sequences per taxon) as a proxy, our data also suggest that growth of other than the typical diatom-associated bacteria [34] appears to have been facilitated (Fig 3). Specifically the phyla *Betaproteobacteria* (*Oxalobacteraceae*, *Comamonadaceae*, *Neisseriaceae*, *Nitrosomonadaceae)*, *Firmicutes (Streptococcaceae*, *Lactobacillaceae*, *Clostridales incertae sedis XI)*, and the candidate division TM7 demonstrated a more than 30% increase in their sequence abundances in HBM compared to LBM sediment (S4 Table). *Bacteroidetes* and *Gammaproteobacteria*, the two phyla that are already known to contain taxa able to assimilate diatom-derived EPS [27] as well as to thrive in the presence of PUAs [78], were dominant in both sediment types (if unclassified phyla are ignored), but specific known diatom-associated bacterial genera [34] within both phyla (*Pseudoalteromonas*, *Maribacter* and *Winogradskyella)* were only represented by few sequences (<40 sequences) in HBM samples. The only exception was *Pseudomonas* [34] well represented (appr. 5000 sequences) and with an increase of over 30% in its relative abundance in HBM sediments. However, considering the relative increase in sequence abundance, *Pseudomonas* and the above mentioned taxa could not have been solely responsible for the observed tenfold increase in total bacterial abundance. To our surprise, despite all these pronounced differences between both sample types, the Bray-Curtis index for community structure remained unaltered due to the dominant influence of relative abundances of abundant OTUs.

Temporal and spatial heterogeneity can be considerable at the micro- and mesoscale in natural environments, and especially in marine sediments [79], stressing the importance of replication. Biological replicates are often pooled before analyses, resulting in loss of spatial and experimental variability [80]. In our study, replicate environmental samples were not pooled and a remarkable degree of variability between replicates of a single sediment type was observed, sharing less than 50% of their OTUs (Fig 2A and 2B). These differences probably result from the patchiness of the sediments that were sampled and/or incomplete sequencing, indicated by the 16S rRNA gene rarefaction curves, resulting in a snapshot of a subset of especially non-abundant taxa. Unfortunately, due to technical issues only two replicates per sampling site in the 16S rRNA gene sequences analysis were analysed, resulting in a considerable loss of information. Next to patchiness, this variability might also result from stochastic processes as explained by the neutral community assembly theory [81]. Despite containing bacteria belonging to different OTUs, calculated richness and diversity indices were similar for different replicates in both sample types (Table 2), which is in agreement with Bowen et al. [82] who reported similar estimates of richness and diversity when comparing individual and homogenized replicate sediment samples of a salt marsh. Only 14–21% of OTUs were shared between both sediment types, again with the non-abundant fraction being responsible for most of the differences in community members (Fig 2, Table 3). Abundant and non-abundant fractions of bacterial communities clearly have a distinct influence on community composition analysis with the non-abundant fraction as major determining factor for phylogenetic diversity [83]. The potential role of these non-abundant taxa remains unclear although it has been suggested that these non-abundant taxa are disproportionally active in comparison to more abundant taxa [81, 84] or can serve as a microbial seed bank [85, 86].

The two-fold increase in MPB biomass was accompanied by an ten-fold increase of denitrifier abundances, while community structure remained unchanged. *NirK* and *nirS* gene abundances correlated well with chl *a*, Si and EPS concentration. The only available comparable study also showed a positive correlation of MPB biomass with *nirK* but not with *nirS* [87], albeit at much lower concentration of chl *a* (ng instead of μg/g dry weight) than here. Interestingly, *nirK* and not *nirS* abundances correlated with levels of organic matter, despite this feature not being significantly different among HBM and LBM samples. This observation confirms differential responses to environmental variables by both *nir* communities [17, 88], and organic matter as important driver of *nirK* denitrifier abundances [89, 90]. Other differential drivers (temperature, salinity and concentration of O_2_, NO_3_^-^, NO_2_^-^, NH_4_^+^) of denitrifier guilds composition [17, 87, 91–94] could not be confirmed here because of absence of correlation or the spatial proximity of the sediment types. In congruence with the few other reports on denitrifier marker genes in estuarine sediments [10, 87, 95], *nirS* genes were one order of magnitude more abundant than *nirK* genes, regardless of sample type. Nevertheless, establishing the *in situ* importance of either *nir* populations in this system requires further studies acquiring gene transcript data and activity measurements [84, 85, 96, 97].

Our *nir* sequences had a close match with sequences from estuarine and marine sediments [20, 95, 98–101] as well as from soil and activated sludge [102, 103], suggesting that the Westerschelde estuary has both tidal and terrestrial influences. They were affiliated with sequences derived from *Alpha*-, *Beta*- and *Gammaproteobacteria* which is similar to previous studies using the same primer sets [104, 105]. However, we need to keep in mind the limitation of the PCR based approach to evaluate denitrifier diversity and abundance. It is widely recognized that available primer sets only target part of the denitrifier population because of high sequence divergence in these molecular markers [104, 106, 107]. With a rough calculation of the potentially undetected functional diversity for *nirK* based on data from the present study we want to put this relevant but often ignored issue into context. All retrieved *nirK* sequences (assigned to approximately 60 OTUs, see Table 2) were related to those from *Alpha*- and *Betaproteobacteria*, with both taxa together responsible for a little over 6% of the total bacterial community diversity (based on number of 16S rRNA OTUs). Let’s assume the following: (i) all *Proteobacterial nirK* OTUs were detected (although this seems highly unlikely considering the reported difference in amplification success among denitrifiers, even within the same genera [108, 109] and reports of *Delta*-, *Gamma*- and *Epsilonproteobacteria* harbouring *nirK* which were not picked up in this study), (ii) other phyla harbouring *nirK* denitrifiers are restricted to *Firmicutes* and *Bacteroidetes*, accounting for over 12% of the community diversity (whole genome studies confirm that denitrifiers from both phyla almost exclusively contain *nirK* but also demonstrate that other phyla do as well), and (iii) the ratio denitrifiers/non-denitrifiers is stable over all phylogenetic lineages, then the undetected *nirK* denitrifier diversity is actually double of what we could detect (120 OTU undetected vs 60 detected). So at best, our study considered one third of this specific denitrifier guild when addressing our research questions. Currently, only shotgun metagenomics can completely uncover *in situ* functional diversity and overcome the limitations of PCR-based sequencing which is essential for further identification of the actual key denitrifiers. Bioinformatic approaches to resolve the bottleneck of detecting target genes in short-read metagenomic libraries are currently under development [110] making it a valid alternative for future phylogenetic studies.

In conclusion, our study indicated that a disproportional tenfold increase in bacterial cell numbers in the sediments coincided with a doubling in biomass of MPB. A similar correlation between MPB biomass and both *nirK* and *nirS* denitrifiers is evidence for the lack of competition for nitrate between denitrifiers and MPB and suggests that MPB are a general determinant of bacterial populations. No causal relation between MPB biomass and bacterial abundance could be inferred as no plausible environmental variable accounting for the doubling in MPB biomass could be deduced from this dataset. Surprisingly, although bacterial abundance did increase tenfold, no significant differences in total bacterial community structure between both sediment types could be detected.

## Supporting Information

S1 Fig Oxygen profiles showing oxygen penetration depth and depth of oxic-anoxic border in HBM and LBM sediment samples (n = 3). (EPS)

S2 Fig Rarefaction curves of the 16S rRNA gene sequences plotting the number of OTUs observed at 3% sequences divergence as function of the number of sequences screened in each replicate. (EPS)

S3 Fig Rarefaction curves for *nirK* (A) and *nirS* (B) plotting the number of OTUs observed at 18 and 20% sequence divergence respectively as function of the number of sequences screened in each replicate. (EPS)

S4 Fig Relative abundances of (A) *nirK* and (B) *nirS* derived OTUs from HBM (n = 3) and LBM (n = 3) sediment samples. Sequences were assigned to OTUs using sequence dissimilarity tresholds of 18% and 20% respectively. All OTUs with a relative abundance below 1% were grouped.
(EPS)

S5 Fig Maximum likelihood tree of representative *nirK* sequences retrieved from HBM and LBM sediment samples. The tree is based on *in silico* translated amino-acid sequences. One representative per OTU is shown. Environmental sequences were included from marine sediments (black dot), soil (grey dot), urethral sample (discontinuous black circle), activated sludge (black circle) and MFC cathode (grey circle). Sequences only represented by an accession number are unknown environmental sequences. Values in parentheses represent the number of sequences that are present from that OTU in a particular replicate. Red colored boxes indicate sequences affiliated to *Alphaproteobacterial nirK* sequences and the blue box represent sequences affiliated to *Betaproteobacterial nirK* sequences. The percentages of replicates trees (n = 1000) in which the OTUs clustered together, are shown next to the branches. Bootstrap values below 50% are not shown.
(EPS)

S6 Fig Maximum likelihood tree of representative *nirS* sequences retrieved from HBM and LBM sediment samples. The tree is based on *in silico* translated amino-acid sequences. One representative per OTU is shown. Environmental sequences were included from marine sediments (black dot), soil (grey dot), activated sludge (black circle), MFC cathode (grey circle) and a membrane reactor (striped dot). Sequences only represented by an accession number are unknown environmental sequences. Values in parentheses represent the number of sequences that are present from that OTU in a particular replicate. Red colored boxes indicate sequences affiliated to *Alphaproteobacterial nirS* sequences, blue boxes represent sequences affiliated to *Betaproteobacterial nirS* sequences, and the green box represents sequences derived from *Gammaproteobacteria nirS* sequences. The percentages of replicates trees (n = 1000) in which the OTUs clustered together, are shown next to the branches. Bootstrap values below 50% are not shown.
(EPS)

S1 Table Complete primer sequences used for 454 pyrosequencing consisting of the adaptor sequence capable of binding to the Lib-L DNA capture beads used for unidirectional sequencing, a key, a particular multiplex identifier (MID) and the primer. (XLSX)

S2 Table Temperature-time profiles for primers used in qPCR of 16S rRNA, *nirK* and *nirS* genes. (XLSX)

S3 Table Correlation matrix of the 16S rRNA, *nirK* and *nirS* gene abundances with environmental parameters. Product moment correlation coefficients are given, significant correlations are indicated in bold. Millimeter depth till oxic-anoxic border and pH were excluded from correlation analyses as no biological replicates were taken for these parameters.
(XLSX)

S4 Table The number of OTUs and sequences of specific taxa found to increase when MPB biomass doubled. The number of OTUs and sequences in HBM and LMB samples are given.
(XLSX)
